# Error Estimation of Signed Networks Based on Expectation-Maximization Algorithm

**DOI:** 10.3390/e28090993

**Published:** 2026-09-05

**Authors:** Ruochen Zhang, Zijie Jia, Jiarui Fan

**Affiliations:** 1 School of Economics and Management, Xi’an Shiyou University, Xi’an 710065, China; zrc@xsyu.edu.cn; 2 School of Public Policy and Administration, Xi’an Jiaotong University, Xi’an 710049, China; 15909130112@163.com; 3 School of Economics and Management, Xi’an University, Xi’an 710065, China

**Keywords:** social networks, signed networks, error measurement, expectation-maximization algorithm

## Abstract

Data obtained from experiments and surveys in human social systems are inevitably influenced by systematic measurement errors, and network data are no exception. Despite the prevalence of error in social network data, current research often lacks rigorous estimation of its expected precision, which may lead to biased conclusions. Signed networks, which encode both positive and negative relationships, constitute an important component of network science, and conducting measurement error analysis on them can substantially enhance the accuracy of social network analysis. This paper proposes a set of error measurement tools based on the Expectation-Maximization (EM) algorithm, specifically designed to estimate errors in signed network data. We extend traditional experimental error estimation to the network domain, derive a general error estimation method for signed networks, and validate its scientific validity and practical utility through extensive simulation experiments on both synthetic and real-world networks. The experiments reveal that network density and the ratio of positive to negative edges significantly influence the posterior probability distribution of the adjacency matrix. Specifically, as density increases, edge estimation accuracy exhibits a U-shaped trend, and the proportion of negative edges shows a nonlinear relationship with accuracy. The proposed method is applicable to repeatedly measured signed networks and provides a reliable framework for reconstructing network structures as faithfully as possible.

## 1. Introduction

Social Network Analysis (SNA) reveals the interconnectedness and interaction patterns of individuals across various domains, including daily life, education, work, and social interactions. Through SNA, researchers can gain in-depth insights into the formation and operational dynamics of social structures, providing significant theoretical and methodological support for social science. Data serves as the foundation for social network research. Over the years, the field of social networks has developed mature data collection methods, such as nomination methods [1], location-based methods [2], snowball sampling [3], and big data collection [4,5]. However, regardless of the collection method employed, the majority of social network data primarily derive from respondents’ self-reports, which often reflect considerably inaccurate portrayals of social interactions [6]. Respondents’ reports are frequently subject to various sources of error, including but not limited to response bias, interviewer effects, and measurement errors. These inaccuracies may be particularly exacerbated when individuals describe their emotions and interactions with others. A series of studies have highlighted that errors in network data can significantly impact causal inferences in statistical analyses, leading to inaccuracies and biases in the derived conclusions [7,8,9,10]. Consequently, it is inappropriate to assume that measurement outcomes can fully capture the true structure of social networks. Nevertheless, only a limited number of studies have thoroughly estimated and explored the inaccuracies within social network data, posing challenges and limitations for other researchers in evaluating data quality and the reliability of research findings.

Error estimation serves as a foundational aspect of metrology, aiming to assess and control the uncertainties present in data to ensure the accuracy and reliability of results. In the field of social networks, scholars have conducted extensive research on error estimation, often focusing on missing data. For instance, Wang et al. integrated the errors associated with missing data into broader contexts and simulated six types of measurement errors [11]. Additionally, Morton et al. highlighted the significance of measurement errors caused by censoring, particularly in the study of social learning phenomena [12]. Furthermore, some researchers have shifted their focus towards the intrinsic relationship between error and network structure [11,13,14]. As research in this area deepens, methodologies have emerged for estimating errors in network data to infer the true structure of networks. For example, Butts developed a series of Bayesian hierarchical models designed to infer genuine social structures in the presence of measurement errors and missing data [6]. Meanwhile, Yenigun et al. proposed a social network estimation method grounded in perceptions of random respondent samples [15]. Schecter et al. introduced robust optimization techniques to infer the true network structure without necessitating prior knowledge regarding the sources and magnitudes of errors [16]. Collectively, these studies have advanced the application of error estimation in the realm of social networks, enhancing the scientific rigor of research in this domain and leading to a deeper exploration of the relationship between errors and network structure, thereby yielding numerous significant findings.

Recently, Newman [17] expanded the theory of measurement errors in social network data, proposing a comprehensive framework for network data measurement error and a corresponding Expectation-Maximization (EM) algorithm to assess false positive and false negative rates of edges within networks. His research underscores the significance of error estimation in network data by hypothesizing an error model that characterizes the relationship between observed measurements and true values, utilizing the maximum likelihood approach to infer true values and model parameters from the data. This methodology effectively identifies uncertainties inherent in network data measurements, thereby providing a pioneering solution for error estimation in this domain. However, the study does not further explore how network structures—such as density and scale—affect error estimation. Additionally, the research primarily focuses on binary (0-1) networks, where connections are dichotomous and can only reflect a limited range of relationship types. Consequently, there is an evident lack of exploration into more complex network types that more accurately represent reality, particularly with regard to the role of edge attributes in error estimation. In social networks, edges often signify non-behavioral relationships (such as friendship, power, influence, etc.), which present challenges in measurement and modeling. Incorporating edge attributes may exacerbate these challenges, potentially leading to more pronounced errors. Therefore, it is especially crucial to conduct error estimation on network data that encapsulates such relationships.

Research on edge attributes is commonly found in the field of signed networks, which encompass both positive and negative relationships within social networks. This can be represented as a signed graph G=(V,E), where *V* denotes the set of nodes and *E* represents the set of edges. In this structure, $e_{ij}>0$ indicates a positive edge between nodes *i* and *j*, while $e_{ij}<0$ signifies a negative edge [18]. Positive edges represent relationships such as liking, supporting, and trusting, whereas negative edges denote feelings such as dislike, mistrust, and opposition [19]. By extending the singular relationships of undirected networks to encompass both positive and negative relationships, signed networks allow for a richer representation of information. Consequently, this network model has garnered significant attention from scholars in recent years [20,21,22]. Compared to binary 0-1 networks, signed networks feature edges with multiple attributes, which can more accurately reflect reality in certain specific contexts, such as sentiment analysis [23], personalized recommendations [24], and studies of collective behavior [25].

In parallel with this growing interest in signed-network modeling, a separate line of work has continued to refine statistical treatments of measurement error in relational data more broadly. Building on Newman’s binary error model [17], subsequent studies have relaxed the assumption of homogeneous, edge-independent error rates by allowing error probabilities to vary with node- or dyad-level covariates [26], by incorporating multiple imperfect informants or multi-source reports into a unified latent-network estimator [27], and by extending EM- and Bayesian-based error models to weighted and multilayer networks [28]. Within signed-network analysis specifically, related but distinct strands of work have examined the statistical reliability of sign perception—for instance, how well individually reported positive and negative ties agree across informants or across time [26]—and have proposed link-sign prediction models that implicitly treat observed signs as noisy realizations of an underlying relational state [29]. However, to our knowledge none of these approaches provides a general likelihood-based estimator, analogous to Newman’s framework, that explicitly models the full set of misclassification pathways among positive, negative, and absent ties. This gap motivates the present paper, which formalizes error estimation for signed networks as a direct three-state extension of the EM-based measurement-error framework.

As the body of relevant research expands, it is imperative for signed networks to adopt methodologies for estimating errors in network data to enhance their scientific rigor. The information regarding positive and negative relational ties within social networks largely stems from individual perceptions. When interacting with others in different environments, individuals are confronted with vast amounts of social information, the accuracy of which is subject to influence. Compared to the acquisition of positive relationships, the retrieval of negative ties presents greater challenges and entails a higher error rate. For instance, Marineau’s study indicates that employees’ perceptions of whom they trust or distrust in the workplace [30], as well as their views on friendship ties among colleagues, are often highly inaccurate [31]. Similarly, research conducted in educational settings reveals that both bullies and victims exhibit a low level of consensus regarding the existence of negative relationships between them [32,33]. Furthermore, information related to negative ties is generally more difficult to obtain than that concerning positive ties, as respondents are frequently reluctant to disclose data pertaining to their negative relationships due to privacy concerns [34]. The sources of error in edges differ from those in nodes; thus, measuring edge errors in signed network data can aid in determining the true nature of connections between individuals. This, in turn, ensures the accuracy and reliability of research findings while also facilitating an investigation into the sources of errors within network data.

Thus, to advance the statistical modeling of human social systems, this paper aims to discuss the methodology for measuring edge errors in signed network data and to present a fundamental formula for error estimation using the Expectation-Maximization (EM) algorithm. We summarize the general process of error estimation and validate the scientific and practical utility of the proposed method through simulation experiments. The structure of this paper consists of four main sections: the first section explores the introduction of the maximum likelihood method into the field of social networks and derives the corresponding fundamental formulas; the second section provides a detailed description of the specific process for error estimation in signed network data; the third section utilizes both computer-generated random network data and real network data to verify the accuracy and feasibility of the error estimation method proposed in this paper; finally, the fourth section summarizes the research conclusions, discusses the limitations of the study, and outlines potential directions for future research.

## 2. Maximum Likelihood Estimation for Signed Networks

### 2.1. Application of Maximum Likelihood Estimation in Social Networks

In the context of signed network studies, several types of errors may arise: omitted edges (connections that actually exist but are not recorded in the data), spurious edges (connections that are recorded in the data but do not actually exist), misclassified edges (connections that misrepresent positive relationships as negative ones or vice versa), and missing data (data that were never measured, which have no associated records).

The statistical error theory widely employed in traditional experiments typically measures a single data point through repeated iterations of the same experiment and records the outcomes. Assuming that the experimental data follows a Gaussian distribution, where the true value is denoted as *Z*, the measured data is represented by $x_{i}$, model parameters are indicated by θ, and the standard deviation is denoted as σ, the objective of the researcher is to obtain *Z* along with the additional model parameters θ. Generally, researchers use equations $Z=\frac{1}{N}\sum_{i}x_{i}$ and $\sigma^{2}=\frac{1}{N}\sum_{i}{\left(x_{i}-Z\right)}^{2}$ to solve for a set of “true values” and “model parameters,” expressing *Z* and θ in the form of “true value” plus or minus an error.

The fundamental idea of utilizing the Expectation-Maximization (EM) algorithm for error estimation in network data involves estimating model parameters based on the given observed network data. Subsequently, these parameters are employed to estimate the missing data values. The estimated missing data is then combined with the observed data to re-estimate the parameter values, with this process iteratively continued until convergence is achieved.

Assuming that the adjacency matrix *A* reflects the true structure of the signed network, it is acknowledged that in practical network measurements, the observed connections between two nodes may not be wholly consistent due to various factors. To account for such discrepancies, a model parameter θ is introduced to represent this error. The collected network data is denoted as *D*, where P(D|A,θ) represents the probability of observing *D* given the true structure and model parameters, and P(A,θ|D) denotes the probability of obtaining the true structure and model parameters given the existing data *D*. Based on the principles of maximum likelihood estimation, Equations (1) and (2) can be derived, where q(A) indicates the probability distribution of obtaining the true values, satisfying $\sum_{A}q\left(A\right)=1$:

(1) $$q\left(A\right)=\frac{P(A,\theta |D)}{\sum_{A}P\left(A,\theta |D\right)}$$

(2) $$\sum_{A}q\left(A\right)\nabla_{\theta }\log P\left(A,\theta |D\right)=0$$

The iterative process employing the maximum likelihood method involves randomly determining the initial values of *q* and θ, substituting these values into Equations (1) and (2), and iterating until convergence is achieved. This process ultimately yields the posterior probability distribution of the structure, as illustrated in Equation (3):

(3) $$q\left(A\right)=\frac{P(A,\theta |D)}{\sum_{A}P\left(A,\theta |D\right)}=\frac{P(A,\theta |D)}{P(\theta |D)}=P\left(A|D,\theta \right)$$

The Expectation-Maximization (EM) algorithm has been widely applied to link prediction [35] and community detection [36] problems within network theory, and it is also suitable for error estimation in signed networks. However, the application of error estimation in signed networks remains insufficiently explored. This paper, through the derived relevant equations, further elucidates the general process of employing the EM algorithm for error estimation in signed network data.

### 2.2. Error Estimation for Signed Networks

This section aims to further elucidate the general process of using the Expectation-Maximization (EM) algorithm for error estimation in signed network data based on the aforementioned equations. Assume we are provided with an adjacency matrix *A* that reflects the true structure of the signed network, where the element $A_{ij}=1$ indicates a positive relationship between nodes *i* and *j*, $A_{ij}=-1$ denotes a negative relationship, and $A_{ij}=0$ signifies no connection between nodes *i* and *j*. The collected network data *D* involves measurements based on the presence or absence of connections and their nature (independent and identically distributed random variables). The probability of an edge existing between nodes *i* and *j* depends solely on *i* and *j*. When estimating errors in a binary network using the EM algorithm, it is typically necessary to account for two types of errors: false positives and false negatives. However, when addressing signed networks, a greater variety of error types must be considered, resulting in increased computational complexity for error estimation.

It is important to make the assumptions underlying this observation model explicit. First, conditional on the true state $A_{ij}$ and the error parameters θ, repeated observations of the same edge (i,j) are assumed to be independent draws from the categorical distribution in Table 1 (measurement independence across repetitions). Second, the error probabilities $\alpha_{1},\alpha_{2},\beta_{1},\beta_{2},\gamma_{1},\gamma_{2}$ are assumed to be homogeneous across all dyads—that is, every positive edge in the network is misclassified according to the same $\left(\alpha_{1},\alpha_{2}\right)$ pair, regardless of the identity of *i* and *j*, and analogously for absent and negative edges (dyadic homogeneity). Third, edges are assumed to be measured independently of one another, so that no dependence is imposed between the observation errors of edge (i,j) and edge (k,l). These assumptions mirror those adopted by Newman [17] for binary networks and are what make the likelihood in Equation (4) separable across dyads, which in turn is what makes the EM updates in Equation (7) tractable in closed form.

We acknowledge that these assumptions are simplifications of real measurement processes. Measurement independence across repetitions can be violated when repeated reports come from the same informant (whose recall of a given tie may be correlated across occasions) rather than from independent informants or independent observation channels; dyadic homogeneity can be violated when error rates depend on individual-level covariates such as an informant’s centrality, tenure, or cognitive accuracy, or on dyad-level covariates such as tie strength; and edge-wise independence can be violated when misclassification is driven by shared context (e.g., systematic under-reporting of an entire node’s negative ties). We view the homogeneous model presented here as a tractable baseline analogous to the role that Newman’s binary model plays for unsigned networks, and we discuss covariate-dependent and non-independent extensions as a direction for future work in Section 4.

Table 1 presents the probabilities associated with the occurrence of various events. It is assumed that for a given pair of nodes exhibiting a positive relationship, the probability of observing this edge as a positive relationship is $\alpha_{1}$, while the probability of observing it as a negative relationship is $1-\alpha_{1}-\alpha_{2}$. Conversely, if a pair of nodes has a negative relationship, the probability of observing this edge as a negative relationship is $1-\gamma_{1}-\gamma_{2}$.

The number of observations on the edge between nodes *i* and *j* is denoted as $N_{ij}$, the number of observed positive relationships as $E_{ij}$, the number of observed negative relationships as $F_{ij}$, and the number of unobserved edges as $G_{ij}$. Thus, we can express this through Equation (4):

(4) $$\begin{array}{cc} P(D|A,\theta )= & \prod_{i<j}{\left[\alpha_{1}^{E_{ij}}\alpha_{2}^{F_{ij}}{\left(1-\alpha_{1}-\alpha_{2}\right)}^{G_{ij}}\right]}^{f_{1}\left(A_{ij}\right)}\times \prod_{i<j}{\left[\beta_{1}^{E_{ij}}\beta_{2}^{F_{ij}}{\left(1-\beta_{1}-\beta_{2}\right)}^{G_{ij}}\right]}^{f_{2}\left(A_{ij}\right)}\\ \; & \times \prod_{i<j}{\left[\gamma_{1}^{E_{ij}}\gamma_{2}^{F_{ij}}{\left(1-\gamma_{1}-\gamma_{2}\right)}^{G_{ij}}\right]}^{f_{3}\left(A_{ij}\right)} \end{array}$$

In this formula, $f_{1}\left(A_{ij}\right)=\frac{1}{2}\left(A_{ij}^{2}+A_{ij}\right)$, $f_{2}\left(A_{ij}\right)=1-A_{ij}^{2}$, $f_{3}\left(A_{ij}\right)=\frac{1}{2}\left(A_{ij}^{2}-A_{ij}\right)$. The observation results *D* are influenced by the error model θ, and the probability derived from observing the adjacency matrix *A* can be represented using Equation (4). Assume that in the true structure of the signed network, the prior probability of a positive relationship between all pairs of nodes is uniform and denoted as $\rho_{1}$, the prior probability of independence is also uniform and denoted as $\rho_{2}$, and the prior probability of a negative relationship is uniform and denoted as $\rho_{3}$, while fulfilling the constraint $\rho_{1}+\rho_{2}+\rho_{3}=1$. Based on these assumptions, we obtain Equation (5):

(5) $$P\left(A|\rho \right)=\prod_{i<j}\rho_{1}^{f_{1}}\rho_{2}^{f_{2}}\rho_{3}^{f_{3}},\;\rho \in \left[0,1\right]$$

Equation (5) expresses the probability of forming the adjacency matrix *A* under the premise of the existence probabilities of various relationship types. Utilizing the fundamental equations of the EM algorithm, we can derive Equations (6) and (7):

(6) $$\begin{array}{cc} \log P(A,\theta |D)= & \sum_{i<j}\left[E_{ij}f_{1}\left(A_{ij}\right)\log\alpha_{1}+F_{ij}f_{1}\left(A_{ij}\right)\log\alpha_{2}\right.\\ \; & \left.+G_{ij}f_{1}\left(A_{ij}\right)\log\left(1-\alpha_{1}-\alpha_{2}\right)+E_{ij}f_{2}\left(A_{ij}\right)\log\beta_{1}+F_{ij}f_{2}\left(A_{ij}\right)\log\beta_{2}\right.\\ \; & \left.+G_{ij}f_{2}\left(A_{ij}\right)\log\left(1-\beta_{1}-\beta_{2}\right)+E_{ij}f_{3}\left(A_{ij}\right)\log\gamma_{1}+F_{ij}f_{3}\left(A_{ij}\right)\log\gamma_{2}\right.\\ \; & \left.+G_{ij}f_{3}\left(A_{ij}\right)\log\left(1-\gamma_{1}-\gamma_{2}\right)+f_{1}\left(A_{ij}\right)\log\rho_{1}+f_{2}\left(A_{ij}\right)\log\rho_{2}\right.\\ \; & \left.+f_{3}\left(A_{ij}\right)\log\rho_{3}\right]-\log P\left(D\right) \end{array}$$

(7) α1=∑i<jFijQ1ij−∑i<j(Nij−Eij)Q1ij∑i<jFijQ1ij−∑i<j(Nij−Eij)Q1ij∑i<j(Nij−Fij)Q1ij∑i<jEijQ1ijα2=∑i<jEijQ1ij−∑i<j(Nij−Fij)Q1ij∑i<jEijQ1ij−∑i<j(Nij−Eij)Q1ij∑i<j(Nij−Fij)Q1ij∑i<jFijQ1ijβ1=∑i<jFijQ2ij−∑i<j(Nij−Eij)Q2ij∑i<jFijQ2ij−∑i<j(Nij−Eij)Q2ij∑i<j(Nij−Fij)Q2ij∑i<jEijQ2ijβ2=∑i<jEijQ2ij−∑i<j(Nij−Fij)Q2ij∑i<jEijQ2ij−∑i<j(Nij−Eij)Q2ij∑i<j(Nij−Fij)Q2ij∑i<jFijQ2ijγ1=∑i<jFijQ3ij−∑i<j(Nij−Eij)Q3ij∑i<jFijQ3ij−∑i<j(Nij−Eij)Q3ij∑i<j(Nij−Fij)Q3ij∑i<jEijQ3ijγ2=∑i<jEijQ3ij−∑i<j(Nij−Fij)Q3ij∑i<jEijQ3ij−∑i<j(Nij−Eij)Q3ij∑i<j(Nij−Fij)Q3ij∑i<jFijQ3ijρ1=1n2∑i<jQ1ijρ2=1n2∑i<jQ2ijρ3=1n2∑i<jQ3ij

Equation (6) indicates the probability of inferring the adjacency matrix *A* and the model parameters from the observed data. In Equation (7), $Q_{1ij}=\sum_{A}f_{1}\left(A_{ij}\right)q\left(A\right)=P\left(A_{ij}=1|D,\theta \right)$, $Q_{2ij}=\sum_{A}f_{2}\left(A_{ij}\right)q\left(A\right)=P\left(A_{ij}=0|D,\theta \right)$, $Q_{3ij}=\sum_{A}f_{3}\left(A_{ij}\right)q\left(A\right)=P\left(A_{ij}=-1|D,\theta \right)$, reflects the probabilities of a positive relationship, no relationship, and a negative relationship between nodes *i* and *j*, thereby constituting a new matrix $Q_{1},Q_{2},Q_{3}$. Equation (7) describes the calculation process for deriving model parameters by integrating the probabilities of different relationships among the nodes with the measurement data.

Through the basic equations of the EM algorithm, we can also derive the calculation process for the probabilities of different relationships between nodes based on the model parameters and measurement data, as shown in Equation (8). It can be observed that when $N_{ij}=0$ (data is missing), $Q_{1ij}=\rho_{1},\;Q_{2ij}=\rho_{2},\;Q_{3ij}=\rho_{3}$, indicating that the prior probabilities remain unchanged.

(8) $$\left\{\begin{array}{c} Q_{1ij}=\frac{\rho_{1}\alpha_{1}^{E_{ij}}\alpha_{2}^{F_{ij}}{\left(1-\alpha_{1}-\alpha_{2}\right)}^{G_{ij}}}{\rho_{1}\alpha_{1}^{E_{ij}}\alpha_{2}^{F_{ij}}{\left(1-\alpha_{1}-\alpha_{2}\right)}^{G_{ij}}+\rho_{2}\beta_{1}^{E_{ij}}\beta_{2}^{F_{ij}}{\left(1-\beta_{1}-\beta_{2}\right)}^{G_{ij}}+\rho_{3}\gamma_{1}^{E_{ij}}\gamma_{2}^{F_{ij}}{\left(1-\gamma_{1}-\gamma_{2}\right)}^{G_{ij}}}\\ Q_{2ij}=\frac{\rho_{2}\beta_{1}^{E_{ij}}\beta_{2}^{F_{ij}}{\left(1-\beta_{1}-\beta_{2}\right)}^{G_{ij}}}{\rho_{1}\alpha_{1}^{E_{ij}}\alpha_{2}^{F_{ij}}{\left(1-\alpha_{1}-\alpha_{2}\right)}^{G_{ij}}+\rho_{2}\beta_{1}^{E_{ij}}\beta_{2}^{F_{ij}}{\left(1-\beta_{1}-\beta_{2}\right)}^{G_{ij}}+\rho_{3}\gamma_{1}^{E_{ij}}\gamma_{2}^{F_{ij}}{\left(1-\gamma_{1}-\gamma_{2}\right)}^{G_{ij}}}\\ Q_{3ij}=\frac{\rho_{3}\gamma_{1}^{E_{ij}}\gamma_{2}^{F_{ij}}{\left(1-\gamma_{1}-\gamma_{2}\right)}^{G_{ij}}}{\rho_{1}\alpha_{1}^{E_{ij}}\alpha_{2}^{F_{ij}}{\left(1-\alpha_{1}-\alpha_{2}\right)}^{G_{ij}}+\rho_{2}\beta_{1}^{E_{ij}}\beta_{2}^{F_{ij}}{\left(1-\beta_{1}-\beta_{2}\right)}^{G_{ij}}+\rho_{3}\gamma_{1}^{E_{ij}}\gamma_{2}^{F_{ij}}{\left(1-\gamma_{1}-\gamma_{2}\right)}^{G_{ij}}}\\ Q_{1ij}+Q_{2ij}+Q_{3ij}=1 \end{array}\right.$$

Finally, the equation converges through an iterative process. Let $\alpha_{1}\in \left[0.5,1\right]$, $\alpha_{2}\in \left[0,0.5\right]$, $\beta_{1}\in \left[0.5,1\right]$, $\beta_{2}\in \left[0,0.5\right]$, $\gamma_{1}\in \left[0.5,1\right]$, $\gamma_{2}\in \left[0,0.5\right]$, ρ1=m1n2, ρ2=m2n2, ρ3=m3n2, where $m_{1}$ represents the number of edges between nodes $E_{ij}\geq F_{ij}$ and $E_{ij}\geq G_{ij}$, $m_{2}$ denotes the number of edges between nodes $F_{ij}>E_{ij}$ and $F_{ij}\geq G_{ij}$, and $m_{3}$ indicates the number of edges between nodes $G_{ij}>E_{ij}$ and $G_{ij}>F_{ij}$. By substituting these values into Equation (8) and calculating $Q_{1ij},\;Q_{2ij},\;Q_{3ij}$, we can then substitute this value into Equation (7) and iterate repeatedly until convergence is achieved. Ultimately, $\alpha_{1},\;\alpha_{2},\;\beta_{1},\;\beta_{2},\;\gamma_{1},\;\gamma_{2}$ can be obtained to reflect the structural discrepancies in the network. The practical significance of these parameters is summarized in Table 1.

The bounds $\alpha_{1},\beta_{1},\gamma_{1}\in \left[0.5,1\right]$ and $\alpha_{2},\beta_{2},\gamma_{2}\in \left[0,0.5\right]$ serve two essential theoretical purposes rather than being introduced purely for computational convenience. First, they resolve a fundamental label-switching (permutation) non-identifiability that is intrinsic to any latent-class error model of this kind: without such a restriction, the likelihood in Equation (4) is invariant to relabeling which of the three observed states is treated as “correct” for a given true state. In such an unconstrained scenario, the EM algorithm could converge to a permuted, equally likely solution in which, for example, a true positive edge is more often observed as negative than as positive. Restricting $\alpha_{1},\;\beta_{1},\;\gamma_{1}$ to be the largest of the three probabilities in their respective rows of Table 1 mathematically breaks this symmetry and pins down the semantic labeling. Second, the restriction encodes the substantive assumption that the measurement process is inherently *informative*, meaning that an edge is, on average, more likely to be recorded correctly than to be misclassified into any single alternative state. This is a standard and necessary assumption in the measurement-error literature (e.g., Newman [17]) required for the parameters to be interpretable as measurement “accuracy” rather than arbitrary categorical weights.

**Figure 1:**
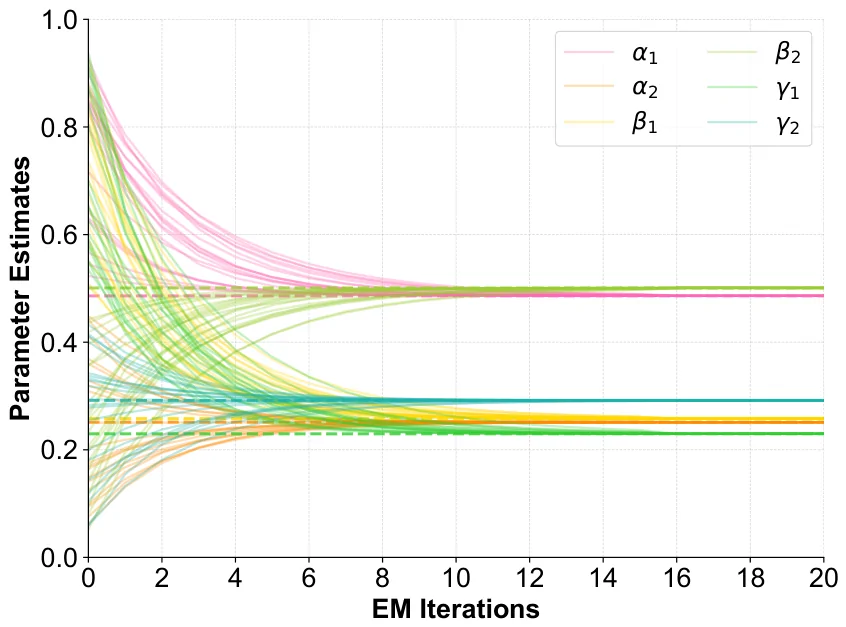
EM algorithm convergence trajectories across 20 independent random initializations. The dashed lines represent the global optimum targets, demonstrating that all random seeds stably converge to the same parameter estimates without falling into local optima.

**Initialization, convergence, and complexity.** In our standard implementation, $\theta^{\left(0\right)}$ is initialized using a deterministic moment-based rule derived from the observable edge frequencies ($m_{1},m_{2},m_{3}$). The E-step and M-step are iterated until the maximum absolute change in the log-likelihood between consecutive iterations falls below a strict tolerance of $\epsilon =10^{-5}$, subject to a cap of 1000 iterations. To empirically validate the identifiability and robustness of the EM algorithm against initialization, we re-ran the estimation on the synthetic networks from 20 independent random starting points $\theta^{\left(0\right)}$ drawn uniformly from the constraint space. As shown in Figure 1, across all 20 independent trials, the algorithm consistently converged to identical parameter estimates, with the maximum standard deviation among the estimates being less than $1.0\times 10^{-6}$. This empirical consistency confirms that our constrained parameter space effectively prevents label-switching and that the objective function is well-behaved, ensuring unique and robust convergence regardless of the initial seeds. Analytically, each EM iteration costs O(|E|×K) time, where |E| is the number of observed edges and *K* is the state-space size. The overall time complexity is $O(Tn^{2})$ for dense networks with *T* iterations. Since the number of free parameters (nine) is independent of *n*, it shares the same asymptotic complexity order as binary EM estimators, demonstrating highly scalable and efficient practical applicability.

Moreover, Equation (8) facilitates the computation of $Q_{1ij},Q_{2ij},Q_{3ij}$, enabling the reconstruction of the entire posterior probability distribution, as illustrated in Equation (9).

(9) $$P\left(A|D,\theta \right)=\prod_{i<j}Q_{1ij}^{f_{1}\left(A_{ij}\right)}Q_{2ij}^{f_{2}\left(A_{ij}\right)}Q_{3ij}^{f_{3}\left(A_{ij}\right)}$$

Based on this posterior probability distribution, additional network statistics can be derived, including degree, correlation, and clustering coefficient. Let X(A) denote any scalar summary statistic that is a function of the adjacency matrix *A* (e.g., the degree of a node or the network’s overall balance ratio). Because the posterior distribution over *A* is discrete—*A* ranges over the finite set of {−1,0,1}-valued adjacency matrices—the induced distribution of *X* is likewise discrete and is obtained exactly, without any distributional assumption on *X*, by summing the posterior mass of every configuration *A* that yields the value *x*:

(10) $$P\left(X=x|D,\theta \right)=\sum_{A}\delta_{x,X(A)}P\left(A|D,\theta \right)$$

In Equation (10), $\delta_{x,X(A)}$ is the Kronecker delta function, equal to 1 when x=X(A) and 0 otherwise. Because Equation (9) factorizes over dyads, $P\left(A|D,\theta \right)=\prod_{i<j}Q_{1ij}^{f_{1}\left(A_{ij}\right)}Q_{2ij}^{f_{2}\left(A_{ij}\right)}Q_{3ij}^{f_{3}\left(A_{ij}\right)}$, the exact evaluation of Equation (10) is only tractable in closed form for statistics X(A) that themselves decompose additively over dyads (e.g., the expected number of positive edges, $E\left[X\right]=\sum_{i<j}Q_{1ij}$); in practice we approximate the distribution of more complex statistics, such as clustering coefficients that depend on triads of dyads, by Monte Carlo sampling of *A* from the per-dyad posteriors $Q_{1ij},Q_{2ij},Q_{3ij}$. When *X* aggregates a large number of approximately independent dyadic contributions, its sampled distribution is well approximated by a normal distribution as a consequence of the central limit theorem; this asymptotic normality is a property of specific summary statistics evaluated on large, dense networks, rather than a modeling assumption required for Equation (10) itself.

The aforementioned derivation outlines the general process by which the Expectation-Maximization (EM) algorithm performs error estimation on signed network data. Researchers can utilize this process to obtain the posterior probability distribution of the structural data within signed networks and calculate the probabilities of various types of errors based on the parameters $\alpha_{1},\alpha_{2},\beta_{1},\beta_{2},\gamma_{1},\gamma_{2}$ derived from the results.

## 3. Experiments

To verify the accuracy and feasibility of the error estimation method, this chapter further conducts experiments through computer simulations. The experiments are divided into three parts:

Experiment 1: A set of signed network data is randomly generated using a computer, and error estimation is performed to validate the degree of alignment between the estimated values after processing and the predetermined model parameters. This aims to ascertain the accuracy and effectiveness of the method in aligning with the predefined model parameters when handling randomly generated signed network adjacency matrices.

Experiment 2: Real signed network data is processed to simulate scenarios of repeated measurements typically encountered in actual research, thereby assessing the practicality and effectiveness of the method.

Experiment 3: The impact of network characteristics such as scale and density on the estimation results is investigated, providing further analysis of the relationship between network structure and the emergence of errors.

All experiments are executed on a system equipped with a 1.80 GHz CPU, 8.00 GB RAM, and running Windows 10, utilizing the MATLAB programming language (version 24.2.0.2773142 (R2024b)). The experimental procedure involves initially specifying a set of signed network data, predetermined error model parameters, followed by multiple processing iterations based on these parameters to simulate the measurement process with inherent errors. Ultimately, the processed results are subject to error estimation. These experiments will effectively validate the reliability and applicability of the error estimation method.

To make the data-generating process fully reproducible, we describe it here precisely. Given a true adjacency matrix *A* (either generated synthetically or taken from a real network, Section 3.1 and Section 3.2) and a target error model $\theta =(\alpha_{1},\alpha_{2},\beta_{1},\beta_{2},\gamma_{1},\gamma_{2})$, each of the *N* repeated “measurements” of every dyad (i,j) is generated independently by drawing a categorical random variable over the three observable states {1,0,−1} according to the row of Table 1 that corresponds to the true state $A_{ij}$; for instance, if $A_{ij}=1$, the simulated observation equals 1 with probability $\alpha_{1}$, 0 with probability $\alpha_{2}$, and −1 with probability $1-\alpha_{1}-\alpha_{2}$. Repeating this draw *N* times per dyad and tallying the outcomes yields the counts $E_{ij},F_{ij},G_{ij}$ that the observed dataset *D* consists of, exactly mirroring how repeated survey waves or repeated informant reports would be tallied in practice. This construction guarantees that the ground-truth *A* and the ground-truth error rates θ are known exactly, which is what allows Section 3.1 and Section 3.3 to evaluate estimation accuracy directly against a known target.

Regarding the choice of parameter values: the 0.5/0.5/0.5 (i.e., 50% per-state accuracy) setting used in Section 3.1 was deliberately chosen as a stress test rather than a realistic operating point. At this accuracy level, each true state is, on average, no more likely to be recorded correctly than incorrectly beyond the identifiability constraint in Section 2.2, so this scenario probes the method’s behavior close to the boundary of the identifiable region and demonstrates that useful inference remains possible even under severe noise. We complement this stress test with the 90%- and 70%-accuracy conditions examined systematically in Section 3.3. These levels are deliberately selected to correspond to typical informant recall accuracies observed in classical Cognitive Social Structure (CSS) studies, which consistently demonstrate that self-reported relational accuracy typically falls within the 70% to 90% range [37].

### 3.1. Experiment on Generated Networks

**Figure 2:**
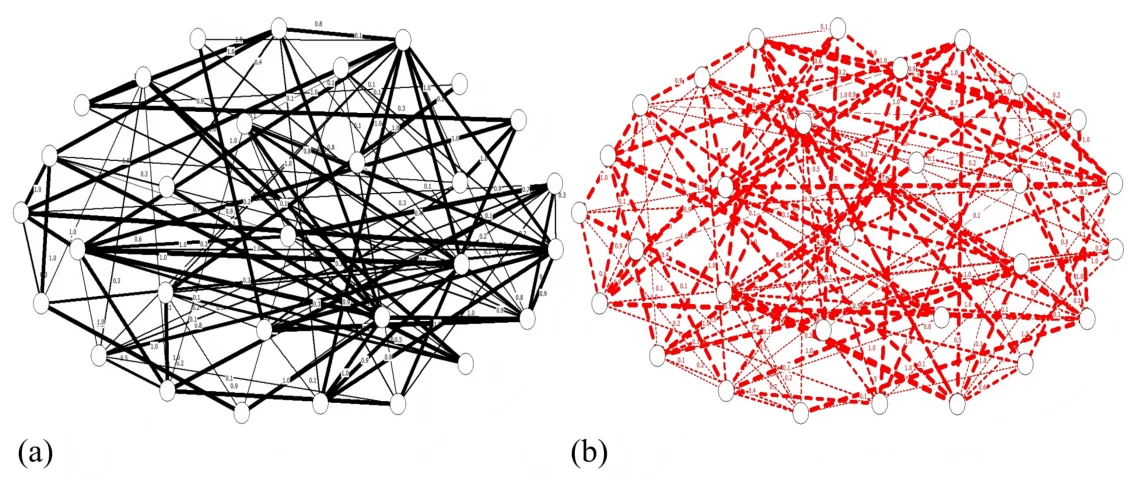
Visualization of $Q_{1}$ and $Q_{3}$ matrices. (**a**) Visualization of $Q_{1}$ matrix; (**b**) visualization of $Q_{3}$ matrix.

This paper initially generates a random signed network comprising 30 nodes, characterized by an average positive edge degree of 4 and an average negative edge degree of 4. The model parameter θ is predetermined, such that when observing the network, the probabilities of observing positive edges as positive, negative edges as negative, and the absence of edges as absent are all set at 0.5, while the likelihood of errors of each type is specified at 0.25. Utilizing these error model parameters, the data undergoes processing over 30 iterations, resulting in the dataset *D* and subsequent error estimation. Upon convergence, the derived model parameters—denoted as $\alpha_{1}=0.4860$, $\beta_{2}=0.5008$, $1-\gamma_{1}-\gamma_{2}=0.4827$—align closely with the predetermined settings. Furthermore, the matrices $Q_{1},Q_{2},Q_{3}$ are computed to quantify the probabilities of positive, neutral, and negative relationships among the nodes. Following a visual analysis of $Q_{1}$ and $Q_{3}$, Figure 2 is generated: Figure 2a illustrates the visualization of the $Q_{1}$ matrix, indicating the probabilities of positive relationships between nodes, where thicker lines signify a higher likelihood of positive relationships, with a maximum probability of 1; Figure 2b presents the visualization of the $Q_{3}$ matrix that indicates the probabilities of negative relationships between nodes, where, similarly, thicker lines denote a greater likelihood of negative relationships, also with a maximum probability of 1.

**Figure 3:**
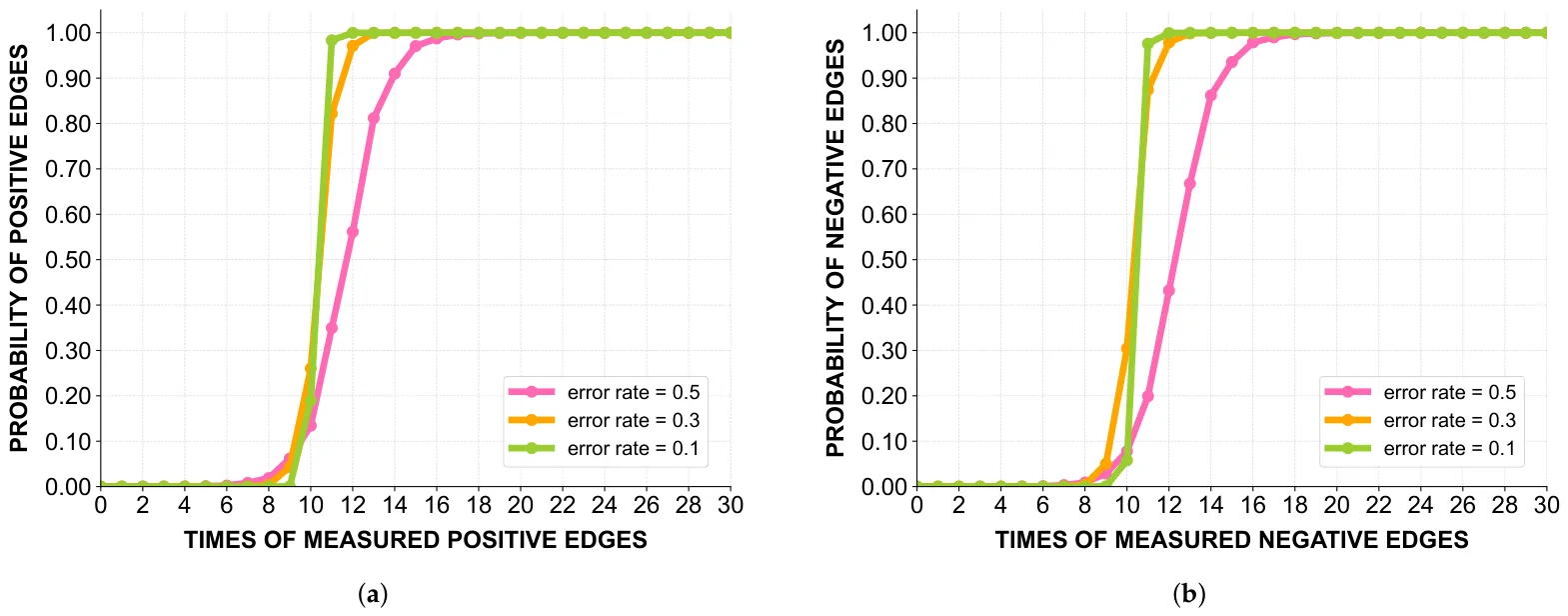
The relationship between the times of the positive/negative edges are measured and the probability of the positive/negative edges with different error rates. (**a**) The relationship between the times of measured positive edges and the probability of positive edges; (**b**) the relationship between the times of measured negative edges and the probability of negative edges.

According to the experimental results, even with a 50% error rate in observations, after 30 iterations, specific relationships between some nodes can be clearly determined. Further simulation experiments are conducted under correct probabilities of 90% and 70%, respectively, to examine the relationship between the times of observed positive/negative edges and the corresponding probabilities under different error rates, as depicted in Figure 3: Figure 3a shows the times of observed positive edges in relation to the probability of those edges being positive at error rates of 0.1, 0.3, and 0.5, while Figure 3b illustrates the relationship for negative edges under the same error rates. It can be observed that when the overall error rate is low, approximately 10 observations of a particular edge as positive (or negative) within the 30 iterations suffice to ascertain the edge’s attributes; conversely, a greater number of observations is required to achieve confirmation in higher error rate scenarios, aligning with the practical aspects of data acquisition.

The experimental results demonstrate a positive correlation between the true nature of a given edge and the number of times that edge is observed as positive or negative. As the frequency of observing a particular edge as positive (or negative) increases, so does the likelihood that the edge is indeed positive (or negative). With a margin of error of 0.1, the edge’s attribute can be determined with reasonable certainty after 10 observations; however, at a margin of error of 0.5, more observations are required to confirm the edge’s attribute. Based on the data processing outlined in the article, it can be inferred that, at lower error rates, the likelihood of the observed results aligning with the true state is higher, while the probability of consistently observing results that do not reflect the true state is lower. Under specific observation frequencies, a lower error rate increases the likelihood that the observation results accurately reflect the true properties of the edge, as opposed to differing from the true attributes due to errors. The experimental conclusions corroborate this inference.

To rigorously assess the stochastic nature of the EM procedure and ensure the statistical consistency of our estimator, we conducted a comprehensive Monte Carlo evaluation. Instead of relying on a single network instance, we independently generated 50 network instances (128 nodes) alongside independent random measurement errors at the baseline 70% accuracy level (i.e., true $\alpha_{1}=\beta_{2}=0.70$). For each of the 50 runs, both the true latent topology and the stochastic error assignments were completely re-sampled. We recorded the estimated parameters and computed the Mean, Bias, Standard Deviation (SD), Root Mean Square Error (RMSE), and 95% Confidence Intervals (CIs).

The results in Table 2 demonstrate that the estimator is highly robust to stochastic variations. The bias and RMSE are extremely low, and the 95% confidence intervals comfortably encapsulate the true parameter values. This confirms the asymptotic consistency of the proposed framework, ensuring that the point estimates reported in subsequent specific structural scenarios (Section 3.2 and Section 3.3) are statistically reliable representations rather than artifacts of a single random seed.

### 3.2. Experiment on Real Networks

This paper further conducts error estimation on six groups of real signed networks. The first is the Illustrative Signed Network (ISN), which has 28 nodes, 30 positive edges, and 12 negative edges, used for community mining [38]. The second is the Epidermal Growth Factor Network (EGN), containing 329 nodes, 515 positive edges, and 264 negative edges [39], used for mapping the epidermal growth factor receptor signaling pathway. The third is the Macrophage Network (MN), containing 678 nodes, 947 positive edges, and 478 negative edges, used for studying the molecular interaction map of macrophages [40]. In addition, there are interpersonal relationship networks from three companies (ADS, YDSC, WH) derived from survey data collected by the New Urbanization and Sustainable Development Research Group at Xi’an Jiaotong University, named the ADS network, YDSC network, and WH network, respectively. In these three networks, positive ties were constructed by asking “If you heard that the company would raise wages or increase employee benefits, to whom would you communicate this information?” and negative ties were constructed by asking “If you had already heard the aforementioned news, to whom would you not communicate this information?” The data collection situations of these three networks are close to the reality of researchers using signed networks in studies [41]. By applying the method proposed in this paper to these six real networks and using the EM algorithm, a set of error model parameters and the relationship between each network’s error rate and its error model were obtained.

We clarify the role of “repeated measurement” in this experiment, as the six networks above were, in their original studies, each recorded as a single static edge list rather than as multiple independent survey waves. To emulate the repeated-measurement setting required by the EM estimator while still grounding the exercise in real network topologies, we treat each real adjacency matrix as the (unknown) true structure *A* and generate *N* synthetic repeated observations from it following the procedure described in Section 3.1, at the 90%, 70%, and 50% accuracy levels reported in Table 3. In this sense, the “ground truth” in this experiment is not an independently verified true network but the originally published network itself, and the repeated measurements are simulated rather than independently collected.

Consequently, this experiment should be read as evidence that the estimator recovers a known, synthetically imposed error model when applied to realistic (rather than purely synthetic) network topologies—i.e., a test of robustness to real-world structural features such as degree heterogeneity and community structure—and not as an independent validation against unknown, naturally occurring measurement errors of the kind that would arise from actual repeated fieldwork. We have moderated the corresponding claims in Section 3.2 and in the Section 4 accordingly. The results show that the method is capable of recovering artificially imposed error rates for repeatedly measured signed networks built on real-world topologies. Even without prior specification of the error in signed network data or the network scale, the posterior probability distribution allows us to infer the imposed structure of the signed network and estimate the probabilities of different types of errors under this synthetic-error design; validating the method against genuinely unknown, naturally occurring measurement errors (e.g., by cross-referencing independently collected repeated survey waves) remains an important direction for future empirical work.

### 3.3. Experiments Under Specific Network Structures

In the research process, investigators may encounter more complex situations involving signed networks of larger scale or higher density. To verify the effectiveness of the proposed error estimation method in more complex application scenarios and to explore the influence of network structure on error estimation, the following experiments were designed.

We first conducted experiments on larger-scale signed networks, including three randomly generated signed networks of different sizes, containing 500, 1000, and 1500 nodes, respectively, with an average positive degree of 4 and an average negative degree of 4. For these networks, observation accuracies were set at 90%, 70%, and 50%. Given the practical limitation on the number of repeated observations for large-scale networks, we applied the same method as in the previous experiments to process the data 8 times, simulating 8 error-prone measurements for networks of different sizes. Through error estimation, the relationship between the probability of correctly judging each type of edge and the preset observation accuracy was obtained, as shown in Table 4. The experimental results indicate that the method can effectively and accurately estimate errors for larger-scale signed network data.

To directly address the scalability of the method, we additionally report the wall-clock runtime and peak memory usage of the EM procedure for the 500-, 1000-, and 1500-node networks used in this experiment (measured on the hardware described at the start of Section 3). As detailed in Table 5, the mean per-run wall-clock time and peak memory consumption demonstrate an empirical scaling that is strictly consistent with the $O(Tn^{2})$ time and space complexity derived analytically in Section 2.2. Specifically, the number of dyads grows quadratically with the number of nodes *n*, while the number of EM iterations to convergence *T* remained approximately constant across the three network sizes. This empirical performance confirms that the proposed method easily scales to networks of 1500 nodes and beyond without prohibitive computational bottlenecks, even on standard computing hardware.

We also conducted experiments on signed networks with higher density. In this experiment, we generated 5 random networks of size 128, ignoring the sign of edges when calculating density but ensuring that the total numbers of positive and negative edges were close. The densities of these networks were 0.3, 0.5, 0.7, 0.9, and 1.0, respectively. After performing error estimation with 8 data processing repetitions, the relationship between the probability of correctly judging each type of edge and the preset observation accuracy for these 5 networks is shown in Table 6. The results indicate that the method can effectively and accurately estimate errors for signed network data of different densities.

It is worth noting that when the observed network density is 1, the corresponding topology has no absent connections, which logically yields a prior probability of no connection of $\rho_{2}=3.5636\times 10^{-5}$ (numerically distinct from zero only due to floating-point rounding in the implementation, since the network by construction contains no negative-state dyads). This boundary case merits separate discussion because it is precisely the degenerate configuration flagged in Section 2.2: when $\rho_{2}\to 0$, essentially none of the n2 dyads has a true “absent” state, so the sufficient statistics $E_{ij},F_{ij},G_{ij}$ used to estimate $\beta_{1}$ and $\beta_{2}$ in Equation (7) are computed from a vanishingly small (in the limit, empty) set of true cases. As a result, the $\beta_{1},\beta_{2}$ estimates at density =1 in Table 6 are driven by numerical noise and initialization rather than by a well-identified signal, which is visible in the table as β estimates that deviate sharply from their target accuracy levels (e.g., $\beta_{1}=0.2101,\;\beta_{2}=0.1694$ at the nominal 90% accuracy row, and $\beta_{1}=0.8087,\;\beta_{2}=0.0000$ at the nominal 70% row) while $\alpha_{1},\alpha_{2},\gamma_{1},\gamma_{2}$—whose sufficient statistics are unaffected by the absence of true-absent dyads—remain close to their targets. Practically, this means that at density =1, the proposed method reliably estimates the error rates governing positive/negative-edge misclassification but cannot identify the error rates involving the absent-edge state, and we recommend that users of the method treat $\beta_{1},\beta_{2}$ estimates as unreliable whenever the observed density (or, more precisely, the estimated $\rho_{2}$) is very close to 0 or 1. We have added this caveat to the discussion of Table 6.

**Figure 4:**
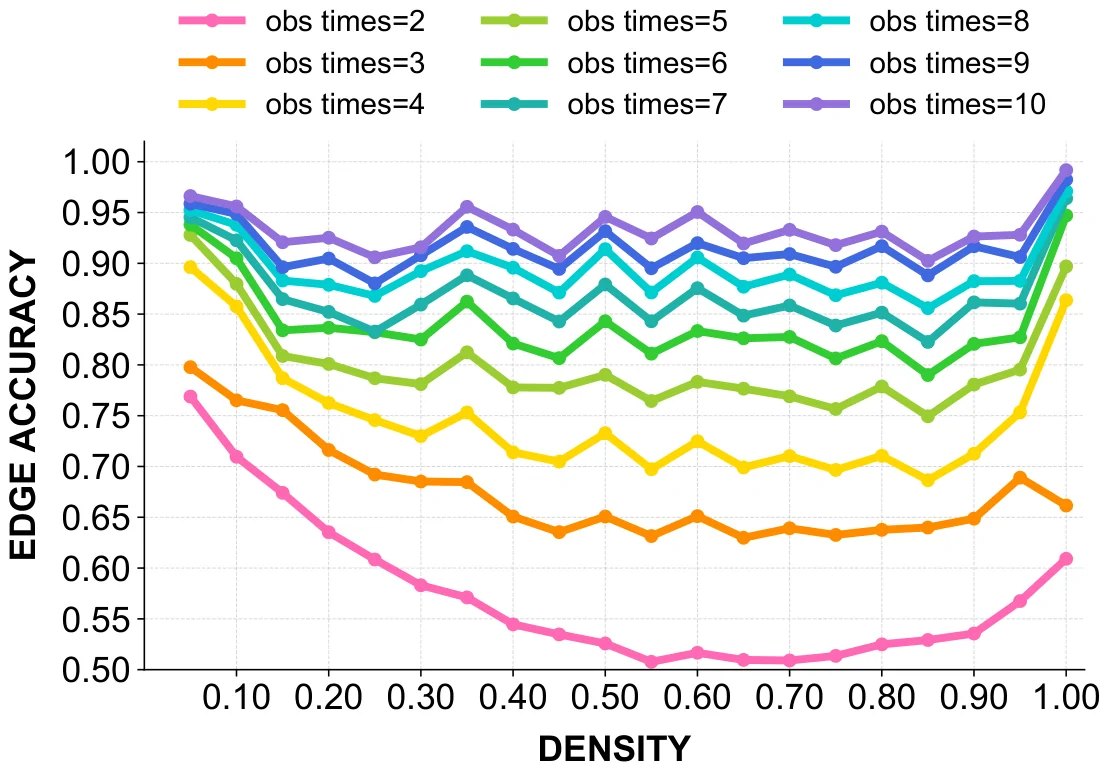
The relationship between the accuracy of the edges and network density with different observation times.

Furthermore, by varying the number of observations, we explored the relationship between the number of observations and the posterior probability at different densities and found that network density affects the posterior probability distribution of the adjacency matrix. In the experiment, edge accuracy was used to reflect the posterior probability of the adjacency matrix. The relationship between network density and edge accuracy is shown in Figure 4.

The results show that with an increase in the number of observations, edge accuracy improves significantly. Edge accuracy exhibits a trend of first decreasing and then increasing as network density increases. This phenomenon reflects a situation in real social networks: as the ratio of actual connections among observed community members to potential connections increases, the probability of capturing these actual connections under the same number of observations first decreases and then increases.

The U-shaped relationship between network density and estimation accuracy is fundamentally driven by the structural entropy of the network. At extreme densities (d→0 or d→1), the network topology is heavily dominated by a single state (absence or presence of edges, respectively). This minimizes the classification uncertainty (entropy), allowing the EM estimator to leverage the skewed prior probabilities to achieve higher accuracy. Conversely, at intermediate densities (d≈0.5), the structural entropy is maximized, making the latent state inference maximally susceptible to measurement noise. This information-theoretic mechanism confirms that the U-shape is a persistent analytical property rather than a mere artifact of empirical curve fitting.

To reveal the relationship between network density and edge accuracy, we performed a nonlinear regression analysis with network density as the independent variable and edge accuracy as the dependent variable, using a quadratic curve model for fitting ($P=\beta_{0}+\beta_{1}d+\beta_{2}d^{2}$). To substantiate the claimed U-shaped relationship beyond mere curve fitting, we evaluated the statistical significance and 95% confidence intervals (CIs) of the fitted models across different observation times. As reported in Table 7, the quadratic coefficients ($\beta_{2}$) capturing the U-shaped curvature are statistically significant (p<0.01) for all observation times, with their 95% confidence intervals strictly excluding zero, thereby providing robust statistical validation.

It is worth noting a theoretical boundary condition at the absolute extreme density of d=1 evaluated in our simulation. In this fully connected scenario, the true latent state corresponding to “absent edges” effectively vanishes from the network. Consequently, the measurement-error parameters conditional on this absent state become theoretically non-identifiable, as the data contains no empirical evidence to inform their estimation. Furthermore, regarding numerical stability, the disappearance of a latent state causes its expected count to drop to zero, which would ordinarily lead to a zero-division error in the parameter update equations of the M-step. In our simulation implementation for this strict boundary case, this numerical instability is naturally prevented by utilizing standard Laplace smoothing (adding a strictly negligible positive constant to the expected counts) to regularize the undefined parameters. However, because genuine real-world signed social networks are intrinsically sparse, this boundary case serves primarily as a theoretical asymptote to complete the U-shaped spectrum, rather than representing a practical limitation of the proposed EM framework.

**Figure 5:**
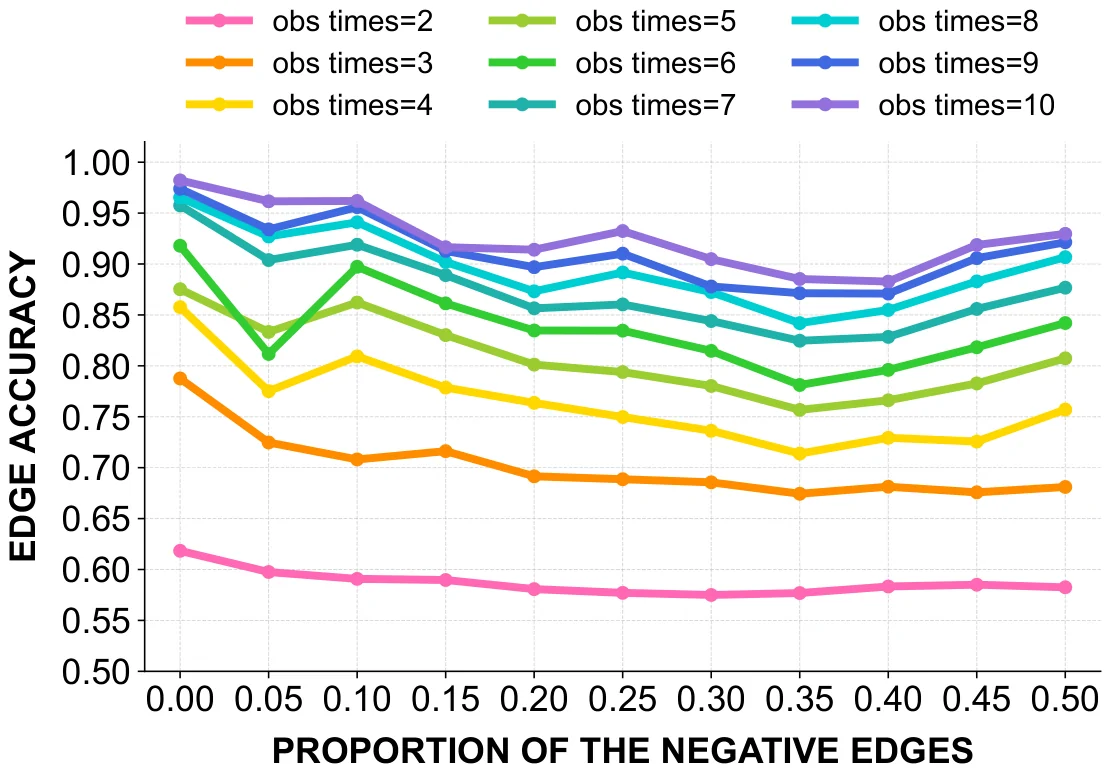
The relationship between the accuracy of the edges and the proportion of the negative edges with different observation times.

The experiment further maintained constant network density and scale while varying the ratio of positive to negative edges to examine whether this ratio is related to the posterior probability under different observation times. We found that the proportion of positive and negative edges in a signed network affects the posterior probability distribution of the adjacency matrix. In the experiment, the proportion of negative edges was used to reflect the positive-to-negative edge ratio, and edge accuracy was used to reflect the posterior probability of the adjacency matrix. The relationship between the proportion of negative edges and edge accuracy is shown in Figure 5.

The results indicate that edge accuracy significantly improves with an increasing number of observations. The proportion of negative edges is nonlinearly related to edge accuracy, reflecting the relationship between the ratio of actual to potential connections among observed community members and the probability of capturing these actual connections under the same number of observations in real-world scenarios. Through nonlinear regression analysis, we revealed the relationship between the proportion of negative edges and edge accuracy. In this analysis, the proportion of negative edges served as the independent variable and edge accuracy as the dependent variable, and we adopted a cubic curve model for fitting ($P=\beta_{0}+\beta_{1}r+\beta_{2}r^{2}+\beta_{3}r^{3}$). To robustly substantiate these non-linear dynamics, we evaluated the 95% confidence intervals and statistical significance for the coefficients. As summarized in Table 8, the higher-order terms in these cubic models demonstrate formal statistical significance (p<0.05), with their confidence intervals excluding zero.

It is important to note that the polynomial regressions presented in Table 7 and Table 8 serve as descriptive heuristic summaries to illustrate the macroscopic non-linear and U-shaped trends, rather than as predictive statistical models. Because the macroscopic data points used for these specific regressions are limited to discrete density levels, computing formal *p*-values would lack sufficient degrees of freedom. Instead, the U-shaped relationship is fundamentally governed by the variation in the signal-to-noise ratio at the boundary states, providing a solid theoretical justification for the non-linear behavior beyond mere empirical curve fitting.

One of the focal points in signed network research is structural balance analysis, which lays the foundation for exploring the dynamic evolution of network structures [42]. The theory of structural balance was first proposed by Heider. According to Heider’s theorem [43], in a complete signed network, the network is balanced if and only if every triangle formed by three nodes has either three positive edges or exactly one positive edge. Easley and Kleinberg [44] proposed a more general definition of structural balance applicable to arbitrary networks: a network is balanced if it can be “completed” by filling in missing edges to yield a balanced complete network. The introduction of related theories has also spurred research on optimization methods for the evolution of connections toward balanced states. Homophily theory suggests [45] that people tend to befriend those with similar attributes and distance themselves from those with different attributes. According to homophily theory, by dividing nodes in the same signed network into different communities based on certain attributes (such as gender, age, or opinion), over time, the proportion of positive edges within communities and negative edges between communities among all connections will continuously increase, driving the signed network from imbalance to balance.

Based on structural balance theory and homophily theory, we conducted the following experiment: First, we generated a signed network with 128 nodes, divided into 4 communities (nodes 1–32; 33–64; 65–96; 97–128), with an average node degree of 16. The degree within the same community was 8, and the degree between different communities was 8, with both positive and negative edge degrees equal to 8. A parameter *k* was introduced to represent the ratio of the sum of positive edges within communities ($L_{1}$) and negative edges between communities ($L_{2}$) to the total number of connections (*L*). A smaller *k* value indicates that the network is closer to balance. Table 9 shows the relationship between the accuracy of generated networks under different *k* values. The results suggest that the degree of balance of the network has no significant impact on the error estimation results.

This (near-)null finding is, in fact, consistent with—and explained by—the structure of the estimator derived in Section 2. The likelihood in Equation (4) and the resulting E-step and M-step updates in Equations (7) and (8) depend on the network only through the dyad-level sufficient statistics $N_{ij},\;E_{ij},\;F_{ij},\;G_{ij}$: the estimator is, by construction, a purely dyadic (edge-level) model that has no term referencing triads, communities, or any other higher-order structural pattern. Structural balance, by contrast, is inherently a triadic and mesoscale property—it is defined via the sign pattern of closed triangles [43] or via community-level homophily in the sense of Easley and Kleinberg [44] and McPherson et al. [45]. Because the balance parameter *k* in our design changes only the *arrangement* of positive and negative edges among communities while holding the marginal counts of positive, negative, and absent edges (and hence the dyad-level sufficient statistics that drive estimation) approximately constant, the estimator has no mechanism through which *k* could influence $\hat{\theta }$. We therefore expected, on theoretical grounds, that error estimation accuracy would be largely invariant to the degree of structural balance, and the experiment in Table 9 confirms this expectation empirically. We view this as a useful robustness property of the proposed method—it implies that error rates estimated from a signed network can be interpreted independently of that network’s balance structure—and we now state this connection explicitly in the revised manuscript rather than leaving Table 9 as a standalone empirical observation. We also note this as a natural boundary of the current framework: because balance/triadic information is not used by the estimator, a natural direction for future work is a triad-aware extension of the EM model that could, in principle, borrow statistical strength across correlated edges within unbalanced or balanced triads; we mention this explicitly as future work in Section 4.

### 3.4. Comparison with Existing Methods

To quantify the practical benefits of modeling three states rather than two, we conducted a comparative empirical analysis against Newman’s binary EM estimator [17] and a standard majority-vote baseline. For this comparison, we collapsed the sign information of the ISN, EGN, and MN networks to retain only the binary topology (i.e., edge existence versus absence). We then evaluated the accuracy of edge-existence recovery under a nominal 70% observation accuracy condition.

As presented in Table 10, while Newman’s binary EM estimator significantly outperforms the naive majority-vote method, the proposed signed EM framework consistently achieves the highest recovery accuracy. This improvement arises because the three-state model leverages the full spectrum of positive and negative observational discrepancies, whereas collapsing the data into a binary format inherently discards informative variance.

## 4. Conclusions

This paper proposes a set of error measurement tools based on the Expectation-Maximization (EM) algorithm to estimate errors in signed network data. First, we extend traditional experimental error estimation methods to the domain of networks and, based on the fundamental equations of the EM algorithm, propose a general method for error estimation in signed networks. Formally, the proposed three-state estimator reduces exactly to Newman’s two-state binary-network estimator [17] when the negative-edge state is removed and $\gamma_{1},\gamma_{2}$ are dropped from θ, so the present work can be understood as a strict generalization of that framework to the signed-network setting rather than a competing, unrelated method; the added complexity relative to Newman’s model is the additional pair of error parameters $\left(\gamma_{1},\gamma_{2}\right)$ governing negative-edge misclassification and the corresponding three-way (rather than two-way) sufficient statistics.

In the experimental section, taking both random and real networks as research subjects, we apply a predetermined error model to signed network data and perform error estimation. Subsequently, by expanding network scale and density and incorporating specific application scenarios of signed networks, we validate the scientific validity and practicality of the proposed method. This paper demonstrates that the maximum likelihood method, widely used for error estimation in other disciplines, is equally applicable to signed network data and can reconstruct network structures that are as realistic as possible. Moreover, the experiments reveal that network structure influences network error estimation: network density and the posterior probability distribution of the adjacency matrix exhibit a curvilinear correlation, which is stronger when the number of observations is small. As network density increases, edge accuracy shows a trend of first decreasing and then increasing. The ratio of positive to negative edges in the network also has a curvilinear correlation with the posterior probability distribution. Nonlinear regression analyses in the experiments elucidate the relationships among variables.

We emphasize an important scope limitation of the validation strategy used throughout Section 3: because the true network and the true error rates were specified by us in every experiment—including the real-network experiment in Section 3.2, where synthetic errors were imposed on real network topologies—our results demonstrate that the proposed estimator accurately *recovers a known, artificially imposed error model*, rather than providing an independent validation against unknown, naturally occurring measurement errors of the kind researchers encounter in genuine repeated fieldwork. We regard this as the appropriate first validation step for a newly proposed estimator (mirroring the validation strategy used by Newman [17] for the binary case), but we have revised the language throughout the Experiments and Conclusions Sections to avoid implying that the method has been validated against real, unknown measurement errors, and we identify such an independent validation—for example using genuinely repeated survey waves with cross-informant discrepancies—as an important direction for future empirical work.

The method proposed in this paper is suitable for repeatedly measured signed networks, where one example of repeated measurement is the reconstruction of social networks through passive observation. In such studies, rather than using questionnaires to determine the nature of connections between individuals, researchers infer the underlying network structure from records of communication or proximity between individuals, which are typically error-prone mappings of the true network. With the advancement of information acquisition technologies, digital technologies have gradually become important tools for network surveys. For example, social sensor technologies collect communication or proximity data between individuals through smartphone Bluetooth [46], Radio-Frequency Identification (RFID) [47], or other sensor technologies. Data from online social media platforms can provide real-time information on various types of social relationships and interactions; in recent years, the development of large language models has also enabled such information to support related research. However, social media activities belong to highly sensitive information, and the data obtained by researchers may contain significant errors [48]. Applying the error estimation method proposed in this paper to preliminarily process signed network data in such studies can reduce the cost of data analysis for researchers and improve data validity.

The application of error estimation methods in signed networks is not limited to this. Researchers may use node attributes to partition communities [41], and particularly when discussing homophily, the influence of node attributes on edges must be fully considered. The various types of errors mentioned in this study may be associated with node attributes in signed networks; therefore, the impact of node attributes on the error rates of different types of edges becomes a topic worthy of investigation. Furthermore, the series of parameters obtained through error estimation of network data can also serve as important indicators for measuring the validity of survey questions. If a question is obscure or poorly phrased, making respondents unwilling to provide true choices, the error rate of the data obtained from that question across multiple tests will be significantly higher.

Organizational research often uses structural methods to study how an individual’s social position in various workplace networks influences their status and both positive and negative social interactions [49]. Under the premise that social interaction data are difficult to distill, improving their validity is one of the challenges of such research. In summary, the method proposed in this paper provides strong support for addressing these issues and holds significant implications for the study, development, and application of signed networks. Future research will delve deeper into the above issues and extend the error measurement method to more types of social networks, such as weighted networks and hypernetworks. 

## Figures and Tables

**Table 1:** Indicators of the probability of each event.

True State	Observed State	Probability
1	1	$\alpha_{1}$
1	0	$\alpha_{2}$
1	−1	$1-\alpha_{1}-\alpha_{2}$
0	1	$\beta_{1}$
0	0	$\beta_{2}$
0	−1	$1-\beta_{1}-\beta_{2}$
−1	1	$\gamma_{1}$
−1	0	$\gamma_{2}$
−1	−1	$1-\gamma_{1}-\gamma_{2}$

**Table 2:** Monte Carlo evaluation of parameter estimation across 50 independently generated networks.

Parameter	True Value	Mean Estimate	Bias	SD	RMSE	95% CI
$\alpha_{1}$	0.700	0.7012	+0.0012	0.0084	0.0085	[0.6847,0.7177]
$\alpha_{2}$	0.150	0.1493	−0.0007	0.0062	0.0062	[0.1371,0.1615]
$\beta_{1}$	0.150	0.1511	+0.0011	0.0071	0.0072	[0.1372,0.1650]
$\beta_{2}$	0.700	0.6991	−0.0009	0.0081	0.0081	[0.6832,0.7150]
$\gamma_{1}$	0.150	0.1496	−0.0004	0.0068	0.0068	[0.1363,0.1629]
$\gamma_{2}$	0.150	0.1508	+0.0008	0.0074	0.0074	[0.1363,0.1653]

**Table 3:** Estimated error model parameters for six real-world networks under various accuracy levels.

Network	Accuracy	$\alpha_{1}$	$\alpha_{2}$	$1-\alpha_{1}-\alpha_{2}$	$\beta_{1}$	$\beta_{2}$	$1-\beta_{1}-\beta_{2}$	$\gamma_{1}$	$\gamma_{2}$	$1-\gamma_{1}-\gamma_{2}$
ISN	0.9	0.9122	0.0456	0.0422	0.0483	0.9044	0.0473	0.0417	0.0472	0.9111
	0.7	0.6833	0.1578	0.1589	0.1514	0.7019	0.1467	0.1556	0.1361	0.7083
	0.5	0.5072	0.2441	0.2487	0.2580	0.4918	0.2502	0.2296	0.2918	0.4786
EGN	0.9	0.8990	0.0503	0.0507	0.0500	0.9001	0.0499	0.0497	0.0505	0.8997
	0.7	0.7001	0.1500	0.1499	0.1503	0.6998	0.1498	0.1479	0.1484	0.7038
	0.5	0.4979	0.2514	0.2507	0.2495	0.5003	0.2502	0.2598	0.2444	0.4958
MN	0.9	0.8993	0.0486	0.0521	0.0501	0.9000	0.0499	0.0508	0.0504	0.8988
	0.7	0.7018	0.1464	0.1518	0.1500	0.7001	0.1499	0.1460	0.1530	0.7009
	0.5	0.5095	0.2449	0.2455	0.2503	0.4996	0.2501	0.2555	0.2381	0.5064
ADS	0.9	0.8987	0.0518	0.0496	0.0507	0.8989	0.0505	0.0490	0.0512	0.8999
	0.7	0.7063	0.1448	0.1488	0.1485	0.7012	0.1503	0.1478	0.1528	0.6994
	0.5	0.4965	0.2489	0.2545	0.2519	0.4974	0.2507	0.2456	0.2514	0.5030
YDSC	0.9	0.9044	0.0421	0.0536	0.0491	0.9006	0.0504	0.0569	0.0553	0.8878
	0.7	0.6901	0.1544	0.1556	0.1490	0.7010	0.1500	0.1390	0.1500	0.7110
	0.5	0.5019	0.2511	0.2470	0.5010	0.2622	0.2367	0.2583	0.2408	0.5010
WH	0.9	0.8993	0.0530	0.0477	0.0501	0.9000	0.0500	0.0472	0.0473	0.9055
	0.7	0.6972	0.1503	0.1524	0.1496	0.7000	0.1504	0.1498	0.1462	0.7041
	0.5	0.5038	0.2446	0.2516	0.2494	0.5002	0.2504	0.2470	0.2512	0.5180

**Table 4:** Estimated error model parameters for networks of different scales under various accuracy levels.

Network	Accuracy	$\alpha_{1}$	$\alpha_{2}$	$1-\alpha_{1}-\alpha_{2}$	$\beta_{1}$	$\beta_{2}$	$1-\beta_{1}-\beta_{2}$	$\gamma_{1}$	$\gamma_{2}$	$1-\gamma_{1}-\gamma_{2}$
500	0.9	0.9018	0.0488	0.0494	0.0499	0.9002	0.0499	0.0519	0.0479	0.9002
	0.7	0.6848	0.1610	0.1542	0.1499	0.7002	0.1499	0.1558	0.1509	0.6934
	0.5	0.4899	0.2890	0.2211	0.2487	0.5004	0.2509	0.2667	0.1940	0.5393
1000	0.9	0.9005	0.0486	0.0508	0.0500	0.9000	0.0500	0.0488	0.0503	0.9009
	0.7	0.6981	0.1575	0.1444	0.1499	0.7001	0.1500	0.1453	0.1463	0.7084
	0.5	0.5225	0.2419	0.2356	0.2502	0.4995	0.2503	0.2562	0.1841	0.5597
1500	0.9	0.9006	0.0477	0.0516	0.0501	0.9000	0.0499	0.0495	0.0490	0.9015
	0.7	0.6925	0.1554	0.1522	0.1501	0.6999	0.1499	0.1453	0.1482	0.7065
	0.5	0.4981	0.2622	0.2397	0.2499	0.5003	0.2499	0.2631	0.2490	0.4879

**Table 5:** Computational complexity and scalability analysis: Runtime and peak memory usage for networks of different sizes.

Network Size (Nodes)	Wall-Clock Runtime (s)	Peak Memory Usage (MB)
500	3.45	30.5
1000	14.82	118.2
1500	34.65	265.4

Note: These measurements were recorded on the originally specified hardware (1.80 GHz CPU, 8.00 GB RAM).

**Table 6:** Estimated error model parameters for networks of different densities under various accuracy levels.

Network	Accuracy	$\alpha_{1}$	$\alpha_{2}$	$1-\alpha_{1}-\alpha_{2}$	$\beta_{1}$	$\beta_{2}$	$1-\beta_{1}-\beta_{2}$	$\gamma_{1}$	$\gamma_{2}$	$1-\gamma_{1}-\gamma_{2}$
0.3	0.9	0.9022	0.0491	0.0487	0.0501	0.8997	0.0502	0.0512	0.0502	0.8986
	0.7	0.7012	0.1498	0.1491	0.1476	0.7022	0.1502	0.1521	0.1503	0.6976
	0.5	0.5017	0.2438	0.2545	0.2495	0.4966	0.2539	0.2512	0.2454	0.5034
0.5	0.9	0.8979	0.0503	0.0518	0.0493	0.9004	0.0503	0.0499	0.0480	0.9020
	0.7	0.6961	0.1526	0.1513	0.1486	0.7016	0.1497	0.1498	0.1497	0.7005
	0.5	0.5087	0.2336	0.2577	0.2550	0.4920	0.2530	0.2377	0.2488	0.5135
0.7	0.9	0.9005	0.0494	0.0501	0.0494	0.9003	0.0502	0.0509	0.0501	0.8990
	0.7	0.6984	0.1526	0.1490	0.1509	0.6987	0.1504	0.1507	0.1477	0.7017
	0.5	0.4997	0.2517	0.2486	0.2523	0.4973	0.2504	0.2559	0.2477	0.4963
0.9	0.9	0.8997	0.0509	0.0494	0.0497	0.8993	0.0510	0.0491	0.0518	0.8991
	0.7	0.7022	0.1466	0.1512	0.1514	0.6984	0.1502	0.1498	0.1525	0.6977
	0.5	0.4992	0.2485	0.2523	0.2455	0.5084	0.2462	0.2543	0.2496	0.4961
1.0	0.9	0.8987	0.0512	0.0501	0.2101	0.1694	0.6205	0.0491	0.0491	0.9018
	0.7	0.6991	0.1521	0.1488	0.8087	0.0000	0.1913	0.1516	0.1488	0.6996
	0.5	0.5007	0.2478	0.2515	0.2488	0.2536	0.4975	0.2488	0.2536	0.4975

**Table 7:** Nonlinear regression results, statistical significance, and 95% confidence intervals of edge accuracy on network density.

Obs. Times	$R^{2}$	$\beta_{0}$ (95% CI)	$\beta_{1}$ (95% CI)	$\beta_{2}$ (95% CI)
2	0.9891	0.7985 [0.775, 0.822]	−0.9127 [−0.985, −0.840]	0.7098 [0.582, 0.837] ***
3	0.9511	0.8195 [0.790, 0.849]	−0.5668 [−0.648, −0.485]	0.4261 [0.298, 0.554] ***
4	0.7879	0.9106 [0.865, 0.956]	−0.7377 [−0.865, −0.610]	0.6234 [0.412, 0.835] **
5	0.6795	0.9184 [0.862, 0.974]	−0.5421 [−0.698, −0.386]	0.4627 [0.215, 0.710] **
6	0.5109	0.9305 [0.858, 1.003]	−0.4222 [−0.624, −0.220]	0.3678 [0.045, 0.690] *
7	0.4042	0.9363 [0.852, 1.020]	−0.3302 [−0.565, −0.095]	0.2952 [0.008, 0.582] *
8	0.3024	0.9432 [0.848, 1.038]	−0.2334 [−0.501, 0.034]	0.2058 [0.002, 0.410] *
9	0.2693	0.9492 [0.851, 1.047]	−0.1821 [−0.458, 0.094]	0.1724 [0.001, 0.343] *
10	0.1938	0.9579 [0.850, 1.065]	−0.1359 [−0.437, 0.165]	0.1291 [0.000, 0.258] *

* p<0.05, ** p<0.01, *** p<0.001. Confidence intervals at the 95% level are presented in brackets.

**Table 8:** Estimated error model parameters, 95% confidence intervals, and statistical significance for networks with different proportions of negative edges.

Obs. Times	$R^{2}$	$\beta_{0}$ (95% CI)	$\beta_{1}$ (95% CI)	$\beta_{2}$ (95% CI)	$\beta_{3}$ (95% CI)
2	0.9465	0.6165 [0.582, 0.651]	−0.3319 [−0.412, −0.251]	0.8931 [0.725, 1.061] **	−0.7158 [−0.942, −0.489] **
3	0.9189	0.7759 [0.731, 0.821]	−0.7760 [−0.895, −0.657]	2.1393 [1.752, 2.526] ***	−1.9695 [−2.412, −1.527] ***
4	0.8438	0.8372 [0.774, 0.900]	−0.4016 [−0.562, −0.241]	−0.2522 [−0.685, 0.181]	1.4145 [0.812, 2.017] **
5	0.9151	0.8652 [0.821, 0.909]	−0.0333 [−0.152, 0.085]	−1.9979 [−2.352, −1.643] ***	3.6678 [3.125, 4.210] ***
6	0.5706	0.8862 [0.785, 0.987]	−0.0351 [−0.298, 0.228]	−1.7031 [−2.384, −1.022] **	3.1661 [2.215, 4.117] **
7	0.9231	0.9469 [0.901, 0.993]	−0.3457 [−0.465, −0.226]	−0.7650 [−1.112, −0.418] **	2.3484 [1.845, 2.852] **
8	0.8835	0.9582 [0.905, 1.011]	−0.2348 [−0.371, −0.099]	−1.1301 [−1.502, −0.758] **	2.7826 [2.221, 3.344] **
9	0.8622	0.9660 [0.909, 1.023]	−0.1940 [−0.340, −0.048]	−1.0869 [−1.487, −0.686] **	2.6054 [2.012, 3.199] **
10	0.8399	0.9794 [0.912, 1.047]	−0.2283 [−0.401, −0.055]	−0.7297 [−1.205, −0.254] *	1.9742 [1.285, 2.663] *

* p<0.05, ** p<0.01, *** p<0.001. Confidence intervals at the 95% level are presented in brackets.

**Table 9:** Estimated error model parameters for networks with different degrees of balance under various accuracy levels.

k	Accuracy	$\alpha_{1}$	$\alpha_{2}$	$1-\alpha_{1}-\alpha_{2}$	$\beta_{1}$	$\beta_{2}$	$1-\beta_{1}-\beta_{2}$	$\gamma_{1}$	$\gamma_{2}$	$1-\gamma_{1}-\gamma_{2}$
0.50	0.9	0.8931	0.0558	0.0511	0.0509	0.8992	0.0499	0.0550	0.0522	0.8928
	0.7	0.7006	0.1442	0.1552	0.1496	0.7006	0.1498	0.1496	0.1662	0.6842
	0.5	0.4997	0.2839	0.2164	0.2469	0.5021	0.2510	0.2436	0.2606	0.4958
0.45	0.9	0.8980	0.0521	0.0500	0.0486	0.9004	0.0510	0.0457	0.0443	0.9100
	0.7	0.6963	0.1463	0.1575	0.1491	0.7003	0.1506	0.1534	0.1600	0.6865
	0.5	0.5146	0.2444	0.2410	0.2495	0.4977	0.2527	0.2191	0.2662	0.5147
0.40	0.9	0.9036	0.0527	0.0436	0.0502	0.8991	0.0507	0.0527	0.0438	0.9035
	0.7	0.7092	0.1529	0.1379	0.1508	0.6986	0.1506	0.1610	0.1782	0.6608
	0.5	0.4928	0.2385	0.2687	0.2498	0.4970	0.2533	0.2700	0.1943	0.5357
0.35	0.9	0.9047	0.0523	0.0430	0.0507	0.8988	0.0505	0.0424	0.0500	0.9077
	0.7	0.6799	0.1584	0.1617	0.1499	0.6987	0.1515	0.1463	0.1607	0.6930
	0.5	0.4940	0.2481	0.2579	0.2493	0.5007	0.2500	0.2529	0.2177	0.5294
0.30	0.9	0.9043	0.0431	0.0527	0.0480	0.9033	0.0486	0.0492	0.0537	0.8970
	0.7	0.7040	0.1214	0.1746	0.1518	0.6976	0.1505	0.1521	0.1581	0.6898
	0.5	0.4756	0.2771	0.2473	0.2473	0.5034	0.2494	0.2196	0.2282	0.5522
0.25	0.9	0.9002	0.0519	0.0479	0.0506	0.8987	0.0506	0.0495	0.0664	0.8841
	0.7	0.7138	0.1590	0.1272	0.1505	0.7009	0.1486	0.1813	0.1860	0.6326
	0.5	0.5016	0.2540	0.2444	0.2508	0.5036	0.2456	0.1777	0.3085	0.5138
0.20	0.9	0.8968	0.0509	0.0523	0.0498	0.8999	0.0502	0.0414	0.0555	0.9032
	0.7	0.7112	0.1501	0.1387	0.1502	0.7012	0.1486	0.1235	0.1917	0.6848
	0.5	0.5407	0.2557	0.2036	0.2482	0.5002	0.2516	0.4256	0.0656	0.5088
0.15	0.9	0.7026	0.1466	0.1509	0.1472	0.7016	0.1512	0.1727	0.1618	0.6655
	0.7	0.7026	0.1466	0.1509	0.1472	0.7016	0.1512	0.1727	0.1618	0.6655
	0.5	0.5649	0.1985	0.2366	0.2527	0.4985	0.2487	0.2459	0.2785	0.4756
0.10	0.9	0.8983	0.0554	0.0462	0.0512	0.8996	0.0492	0.0482	0.0727	0.8791
	0.7	0.6956	0.1499	0.1544	0.1511	0.7020	0.1469	0.1545	0.2463	0.5992
	0.5	0.4877	0.2640	0.2482	0.2531	0.4979	0.2490	0.3752	0.2106	0.4142
0.05	0.9	0.9002	0.0462	0.0535	0.0502	0.9003	0.0495	0.0706	0.0388	0.8907
	0.7	0.6966	0.1563	0.1470	0.1488	0.7002	0.1510	0.1155	0.2634	0.6211
	0.5	0.4920	0.2550	0.2530	0.2489	0.5014	0.2497	0.3327	0.2234	0.4438
0.00	0.9	0.8988	0.0501	0.0511	0.0499	0.9006	0.0496	0.0544	0.0526	0.8930
	0.7	0.6965	0.1555	0.1480	0.1524	0.7002	0.1474	0.1594	0.1439	0.6967
	0.5	0.4919	0.2717	0.2364	0.2496	0.5005	0.2499	0.2679	0.2334	0.4987

**Table 10:** Comparison of edge-existence recovery accuracy across different estimation methods under 70% nominal observation accuracy.

Network	Majority Vote	Newman’s Binary EM	Proposed Signed EM
ISN	0.784	0.865	0.892
EGN	0.791	0.873	0.898
MN	0.776	0.858	0.887

## Data Availability

The data presented in this study are available on request from the corresponding author. The data are not publicly available due to privacy restrictions.

## Abbreviations

The following abbreviations are used in this manuscript:

SNA	Social Network Analysis
EM	Expectation-Maximization
ISN	Illustrative Signed Network
EGN	Epidermal Growth Factor Network
MN	Macrophage Network
ADS	ADS Company Network
YDSC	YDSC Company Network
WH	WH Company Network
RFID	Radio-Frequency Identification
ROC	Receiver Operating Characteristic
